# The influence of thermal stress on the physical and technical activities of soccer players: lessons from the 2018 FIFA World Cup in Russia

**DOI:** 10.1007/s00484-020-01964-3

**Published:** 2020-07-16

**Authors:** Marek Konefał, Paweł Chmura, Michał Zacharko, Jarosław Baranowski, Marcin Andrzejewski, Krzysztof Błażejczyk, Jan Chmura

**Affiliations:** 1 Department of Biological and Motor Sport Bases, University School of Physical Education, I.J. Paderewskiego 35, Wrocław, Poland; 2 Department of Team Games, University School of Physical Education, I.J. Paderewskiego 35, Wrocław, Poland; 3 Institute of Geography and Spatial Organization, Polish Academy of Sciences, Twarda 51/55, Warszawa, Poland; 4 Department Methodology of Recreation, Poznan University of Physical Education, Królowej Jadwigi 27/39, Poznań, Poland

**Keywords:** UTCI, Training centres, Sprints, Passes, Football, Heart rate effort, Evaporative water loss

## Abstract

The present study attempts to assess changes in soccer players’ physical and technical activity profiles due to thermal stress, measured with the Universal Thermal Climate Index (UTCI), in training centres and during matches of the 2018 FIFA World Cup in Russia. The study also verifies the theoretical models of soccer players’ physiological parameters. The study sample consisted of 945 observations of 340 players of national teams taking part in the World Cup in Russia. The measured variables included physical activities: total distance covered, distances covered with an intensity of 20–25 km/h, number of sprints; technical activities: number of shots, number of passes, pass accuracy and physiological indicators: evaporative water loss and heart rate. In addition, the final ranking places of each national team were also used in the study. The UTCI was calculated based on meteorological data recorded at training centres and during matches. The UTCI records were then classified into two ranges: NTS—no thermal stress (UTCI 9–26 °C) and TS—thermal stress (UTCI > 26 °C). Climatic conditions at soccer training centres assessed as involving “no thermal stress” are found to be more beneficial for increasing the total distance covered and the number of sprints performed by players during a match. The theoretical models for determining soccer players’ physiological parameters used in the study reduce the players’ heart rate effort and evaporative water loss, which is in line with findings in studies by other authors. The climatic conditions at soccer training centres and during tournament matches should be taken into account in planning preparations for future World Cup tournaments, especially those in hotter countries.

## Introduction

The improvement of the quality of players’ performance and match outcome in modern association football requires a detailed analysis of various parameters of the game and external factors (Sarmento et al. 2014). Soccer match analysis is currently carried out with the use of technologically advanced motion analysis systems that precisely record and reliably process data on players’ physical and technical activities (Carling et al. 2008; Hoppe et al. 2015). However, due to their diversity, the identification of external factors influencing the game often requires a great deal of interdisciplinary research (Hosokawa et al. 2018). Comprehensive data obtained during the analysis is highly valuable, as they allow the coaching staff to consciously manage their team during the match and effectively plan the team preparation for the competition (Wright et al. 2013).

Biometeorological conditions are such external factors that significantly affect players’ behaviours during a soccer match. However, their precise determination is especially difficult due to the different strength of impact of many ambient environment parameters. The Universal Thermal Climate Index (UTCI), which represents different climates, weather and locations, is one of the many indicators currently used to assess biometeorological conditions for humans (Błażejczyk et al. 2012). Moreover, like the human body, the UTCI is very sensitive to environmental stimuli: temperature, solar radiation, wind and humidity. The UTCI considers the time variability of thermal conditions, and its scale is sensitive to even the smallest differences in the intensity of meteorological stimuli (Błażejczyk et al. 2012).

Winning a soccer match depends on the appropriate level of players’ physical and technical parameters combined with the right team tactics (Carling and Dupont 2011; Mackenzie and Cushion 2013). The distance covered by professional players over a 90-min match usually ranges from 10 to 12 km (Mascio and Bradley 2013; Andrzejewski et al. 2016). The number of sprints performed by a single player in a match can range from 3 to 40; however, this variable is highly individual and depends on, for example, the speed range defining the sprint (Carling et al. 2012; Andrzejewski et al. 2018). Although high-intensity exercises (fast running and sprinting) constitute only about 10–15% of the total distance covered by players during a match (Andrzejewski et al. 2016; Chmura et al. 2018), they are among the most important physical activities of modern footballers (Ingebrigtsen et al. 2015; Struzik et al. 2016; Maćkała et al. 2019). The most frequently measured technical activities of soccer players are the total number of performed shots and shots on target and the number of successful passes (pass accuracy) (Castellano et al. 2012; Lago-Penas and Gomez-Lopez 2014). Studies of the World Cup matches in 2002, 2006, 2010 and 2014 revealed that the efficiency of shots and passes of winning teams was much higher than of losing teams (Njororai 2013; Liu et al. 2015; Smith and Lyons 2017).

When biometeorological conditions were considered in the analysis of soccer players’ physical and technical activities, the high air temperature resulted in a decrease in players’ high-intensity activities during the matches of the 2014 World Cup in Brazil. The total number of sprints performed by soccer players was also significantly lower in both higher temperatures and humidity (Konefał et al. 2014). Subsequent analyses showed that the longest recorded mean distance (10.5 km) was covered by players in an air temperature below 22 °C and relative humidity below 60%, while the shortest (9.8 km) was covered in the same air temperature range, but with relative humidity above 60%. Significant differences in the number of sprints performed by players were found between the air temperature ranges below 22 °C (41 performed sprints) and above 28 °C (31), but only at the relative humidity below 60% (Chmura et al. 2017a). What is more, Nassis et al. (2015) found that while the numbers of passes did not differ, the pass accuracy was higher under high, rather than low, environmental stress conditions.

Biometeorological conditions affect players’ physical and technical activities, which is a consequence of the physiological reactions of players’ bodies (Chapelle et al. 2019). Often the changes in players’ match activity are not only due to the climatic conditions in which the match takes place but also the conditions in the training centre where the team is preparing for the match. As Périard et al. (2015) claim, proper acclimatization is conducive to the physical abilities of footballers. Evaporative water loss and heart rate are important physiological parameters of a player’s activity during a match. Sweat loss in team sports can be significant due to repeated bursts of high-intensity activity, in addition to the fact that environmental heat stress is often present during training and competition. Hypohydration typically impairs performance at higher BML levels (3–4%) (Nuccio et al. 2017). Thermal stress and, consequently, high dehydration levels cause a marked increase in players’ heart rate (Périard et al. 2015). During soccer championship matches, it is not easy to measure players’ physiological parameters on an ongoing basis. Therefore, attempts to mathematically calculate these parameters, and suggestions for various indicators based on other more easily measurable parameters, can be found in scientific literature (Malchaire 1991; ISO/DIS 7933 (2004); ISO 8996 (2004); Parsons 2003; de Freitas and Grigorieva 2017).

A large number of factors influence the match outcome. The differences between the teams playing at the highest level are small, and a successful fight for the World Championship title requires attention to many details. There have been no studies so far on the relationship between the UTCI, players’ physical and technical activities and physiological parameters. The analysis of these parameters in the context of biometeorological conditions in soccer training centres and during matches is innovative. Such research is particularly important for sports practice in the context of the next FIFA World Cup, which will be held in Qatar in 2022, as well for subsequent tournaments. This study can be useful to soccer training staffs working on the directions of optimal physical preparation of players before top-level competitions, which take place in various, and often adverse, climatic conditions.

The present study attempts to assess changes in soccer players’ physical and technical activity profiles due to thermal stress, measured with the Universal Thermal Climate Index (UTCI) in training centres and during matches of the 2018 FIFA World Cup in Russia. The secondary goal of the study was to verify certain theoretical models for determining physiological parameters of soccer players.

## Materials and methods

### Subjects

The study material consisted of 945 observations of 340 soccer players, excluding goalkeepers, representing the 32 national teams taking part in the 2018 FIFA World Cup in Russia. All the participants played full-time matches (without extra time) of the group and knockout stages of the tournament. The players’ mean body height was 182.03 ± 6.90 cm, body mass 77.11 ± 6.99 kg, and age 27.10 ± 3.55 years (https://www.fifa.com/worldcup/archive/russia2018/matches/).

The study was conducted in compliance with the Declaration of Helsinki and was approved by the local ethics committee (No. 19/2017).

### Procedures

#### Biometeorological conditions

The study used the Universal Thermal Climate Index (UTCI) which is calculated on the basis of meteorological data (air temperature and humidity, wind speed, and cloudiness) recorded at the training centres (average of all days between matches) and during the World Cup matches. The gathered UTCI records were classified into two ranges: NTS—no thermal stress (UTCI 9–26 °C) and TS—thermal stress (UTCI > 26 °C). Next, four categories of UTCI (dUTCI) changes were identified (UTCI at the training centres → UTCI during the match): NTS → NTS (*n* = 303), NTS → TS (*n* = 194), TS → NTS (*n* = 236), and TS → TS (*n* = 212). The applied UTCI ranges were based on the results of earlier studies by Błażejczyk et al. (2012) and Jendritzky et al. (2012). Meteorological data necessary for the calculation of UTCI were retrieved from the meteorological stations located nearest the training centres, available from the Russian Weather Service (http://aisori-m.meteo.ru/waisori/) and Ogimet (https://www.ogimet.com/synops.phtml.en) databases. As far as the data from the training centres were concerned, it was assumed that the players’ activities took place mostly around midday hours. Meteorological data were thus also taken for that time of the day. Data on air temperature and humidity during matches were retrieved from the stadiums’ meteorological stations (official FIFA reports, https://www.fifa.com/worldcup/archive/russia2018/matches/match/300331503/#match-info). The calculations were made with the use of BioKlima 2.6 software package (www.igipz.pan.pl/geoekoklimat/blaz/bioklima.htm).

#### Physical and technical activities

The STATS® motion analysis system was used to record players’ movements during all tournament matches. The system provides a kinematic analysis of players’ performance in real time with a set of video cameras. Every possible type of ball touch and on-the-ball action in a match is covered by a rigid set of definitions recorded in the system. Every action requires a player to be assigned to it, along with a time stamp indicating when the event occurred (Liu et al. 2015). The STATS® motion analysis system is a reliable research tool to track players’ movements at top-level football tournaments, such as the European Championships and the FIFA World Cup (Linke et al. 2018). The inter-operator reliability of the tracking system used to collect soccer match statistics was identified to be at an acceptable level (Linke et al. 2018).

The measured indices included players’ physical activities: total distance covered [km], distances covered with an intensity of 20–25 km/h [km] and number of sprints performed and technical activities: number of shots (an attempt to score a goal made with any (legal) part of the body, either on or off target), number of passes (a ball played intentionally from one player to another) and pass accuracy [%] (successful passes as a proportion of total passes). In addition, the final rankings of each national team (from the 1st to 32nd place) were also used. The study was retrospective. Relevant match data for these indices were retrieved from the official FIFA website (https://www.fifa.com/worldcup/archive/russia2018/matches/), gathered using the STATS® advanced motion analysis system (Chicago, IL, USA).

#### Physiological parameters

Two physiological indices of players were also considered. The first was heart rate effort (HR-E) [BPM], i.e. a player’s heart rate during the match (assumed workload of 400 W/m^2^). HR-E was calculated using the Fuller and Brouha (1966) formula:

$$ \mathrm{HR}-\mathrm{E}=22.4+0.18\ \mathrm{M}+0.25\ \left(5\ \mathrm{Ta}+2.66\ \mathrm{vp}\right) $$

where M—metabolic work load (W/m^2^), Ta—ambient temperature (°C) and vp—vapour pressure (hPa).

The other index was evaporative water loss (EVL) [g/h], i.e. water loss through sweat evaporation during the match. The EVL index derives from the Man-Environment heat EXchange model MENEX (Błażejczyk 1994; 2005; Błażejczyk and Kunert 2011). The calculations of both indices were made using the BioKlima 2.6 software package (www.igipz.pan.pl/geoekoklimat/blaz/bioklima.htm).

#### Statistical analyses

Values for all parameters were checked to verify their conformity with a normal distribution (Shapiro-Wilk test). Arithmetic means and standard deviations were also calculated with the means then being compared using one-way analysis of variance (ANOVA). Differences between pairs of means were checked using Fisher’s least significant difference (LSD) test, with the level of statistical significance at *p* ≤ 0.05. All statistical analyses were made using the STATISTICA ver. 13.1 software package (from StatSoft. Inc., USA).

## Results

**Fig. 1:**
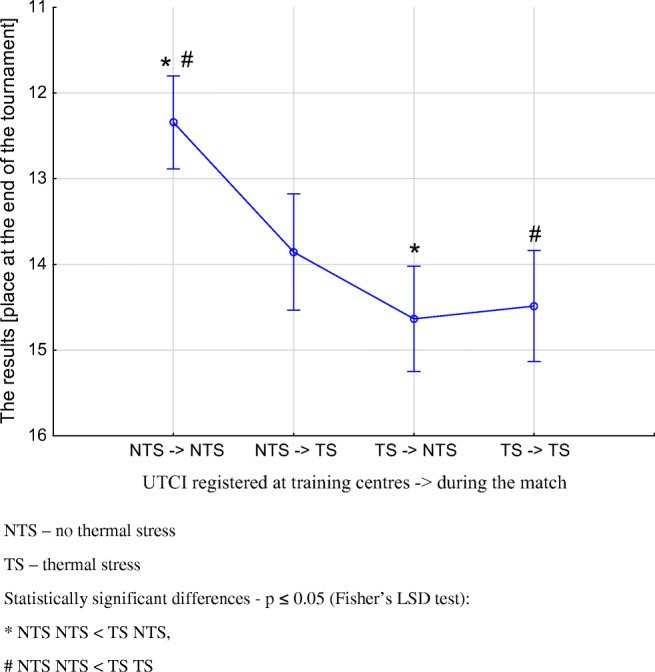
Differences in the place at the end of the tournament with regard to UTCI ranges (mean ± SD). NTS—no thermal stress, TS—thermal stress. Statistically significant differences—*p* ≤ 0.05 (Fisher’s LSD test): *NTS → NTS < TS → NTS, **#**NTS → NTS < TS → TS

The statistical analysis of the study parameters in relation to the dUTCI ranges (NTS → NTS, NTS → TS, TS → NTS, TS → TS) revealed effects with regard to a national team’s place at the end of the tournament ranking (F = 3.371(3); *p* = 0.018) (Fig. 1). Also, the effects were revealed in relation to the total distance covered (F = 30.960(3); *p* = 0.001), distances covered with the intensity of 20-25 km/h (F = 11.929(3); *p* = 0.001), numbers of performed sprints (F = 8.401(3); *p* = 0.001) and numbers of performed passes (F = 2.472(3); *p* = 0.050). In terms of players’ physical parameters, the best results were recorded in NTS → NTS. The analysis of physiological parameters revealed the effects in heart rate effort (F = 278.200(3); *p* = 0.001) and evaporative water loss (F = 308.330(3); *p* = 0.001). Greater parameter values were noted in TS → TS (Table 1).

**Table 1:** Differences in the physical and technical activity engaged in by soccer players with regard to UTCI ranges (mean ± SD)

Parameters	UTCI: registered at training centres → during the match				SSD (*p* ≤ 0.05)
	NTS → NTS (1)	NTS → TS (2)	TS → NTS (3)	TS → TS (4)	
Total distance covered (km)	10.55 ± 1.45	9.73 ± 0.95	10.26 ± 1.37	9.62 ± 0.95	1 > 2.3.4; 3 > 2.4
Distance covered at 20–25 km/h (m)	563.73 ± 188.34	509.40 ± 166.61	542.68 ± 178.63	475.71 ± 153.04	1 > 2.4; 3 > 2.4
Sprints (number)	33.42 ± 12.17	30.77 ± 11.13	31.82 ± 11.39	28.43 ± 9.93	1 > 2.4; 1.2.3 > 4
Shots (number)	1.08 ± 1.34	1.24 ± 1.60	1.17 ± 1.44	1.05 ± 1.35	–
Passes (number)	44.87 ± 19.11	45.41 ± 24.94	47.98 ± 28.34	42.00 ± 21.05	3 > 4
Pass Accuracy (%)	83.17 ± 10.17	83.90 ± 10.61	82.94 ± 9.76	82.47 ± 11.76	–
Heart Rate Effort (HR-E) (BPM)	127.68 ± 6.08	138.46 ± 5.17	129.30 ± 5.88	140.36 ± 6.30	1 < 3 < 2 < 4
Evaporative Water Loss (EVL) (g/h)	522.78 ± 61.17	722.15 ± 140.86	531.65 ± 57.96	793.04 ± 188.72	1 < 2.4; 3 < 2; 4 > 1.2.3

*NTS* no thermal stress, *TS* thermal stress, *SSD* statistically significant differences—*p* ≤ 0.05 (Fisher’s LSD test)

No significant effect was found for the number of shots (F = 0.738(3); *p* = 0.529) and pass accuracy (F = 0.650(3); *p* = 0.584) in relation to the dUTCI category (Table 1).

## Discussion

The use of climate data in organizational decisions related to soccer tournaments is necessary to reduce the potential risk of thermal stress (TS), or to implement plans for optimal preparation of players to compete in adverse biometeorological conditions (Hosokawa et al. 2018). In terms of practical use of this data analysis, changes in soccer players’ physical and technical activity profiles, due to thermal stress, measured by the UTCI at the training centres and during matches are indicated.

Research to date has indicated that the more difficult biometeorological conditions during a competition are, the harder it is to succeed (Mohr et al. 2012, Chmura et al. 2017a). The present study confirms these reports but also makes a novel observation that when matches take place in conditions of no thermal stress (NTS), the biometeorological conditions at training centres also prove to be significant for the match outcome. If soccer players are exposed to NTS before matches, then their teams, on average, occupy a higher overall position by over two places in the ranking of national teams, compared to teams exposed to TS (Fig. 1). In addition, the lowest ranked teams are those exposed to TS, regardless of the conditions in which the next match takes place. It follows that the climatic conditions to which players are exposed during the whole duration of a football tournament are as significant as the particular conditions in which the actual matches take place. However, it should be noted that the training staffs of national teams do not have any influence on match location (chosen on a random basis), whereas they do have a choice of training centre locations for their players. Thus, it seems necessary to be aware of the predicted biometeorological conditions.

Research indicates that the most comfortable conditions for physical activity include air temperature below 22 °C, relative humidity below 60% (Chmura et al. 2017a), wet-bulb globe temperature (WBGT) below 22 °C (Nassis et al. 2015) or the UTCI between 9 and 26 °C, where heat load and thermal stress do not occur (Błażejczyk et al. 2012). As the present study indicates, when a match takes place in such comfortable conditions, players run, on average, between 10.26 ± 1.37 km (TS → NTS) and 10.55 ± 1.45 km (NTS → NTS). It can be observed that comfortable biometeorological conditions at the training centres increase the average distance covered by each player in a match by 290 m, and thus, the distance covered by an entire team by about 3 km. The importance of this physical activity in soccer players is emphasized by the fact that on average, each player in the German national team—which won the World Cup in 2014—covered 10.39 ± 1.19 km per match. This was significantly higher (340 m) than the distance covered by players from other teams participating in the tournament (Chmura et al. 2017b). Moreover, Andrzejewski et al. (2018) found that winning a match is correlated with the players of the victorious team covering a longer total distance.

The results of our research show that the shortest total distance (9.62 ± 0.95 km), distance covered at 20–25 km/h (475.71 ± 153.04 m), and the lowest number of sprints (28.43 ± 9.93) were recorded in TS. This is not surprising given that the existing literature indicates that even elite soccer players notice the negative impact of adverse biometeorological conditions on their physical activity during a match (Özgünen et al. 2010; Hayes et al. 2014; Link and Weber 2017). The revealed differences in soccer players’ physical activity at 18 °C vs 30 °C and at 21 °C vs 43 °C indicate that when the exercises were performed in a hot environment, the total distance covered was up to 9% lower, the number of sprints performed − 10% lower, and the distance covered with a high intensity 26% shorter (Mohr et al. 2012; Konefał et al. 2014; Aldous et al. 2016). However, the fact of a significantly greater number of sprints performed by players in NTS → TS (30.77 ± 11.13) than in TS → TS (28.43 ± 9.93) found in the present study brings is a novel observation of soccer players’ activity during matches taking place in TS. Indeed, it shows that when teams and individual players have to deal with difficult game conditions, biometeorological conditions at the training centres also affect the performance of players’ activities of very high intensity. This is significant information for the coaching staffs as many authors indicate that the distance covered with a high intensity is a key physical parameter affecting the match outcome (Faude et al. 2012). Using logistic models based on the German Bundesliga matches, Konefał et al. (2019a) showed that increasing the distance covered at a very high intensity by 0.1 km was associated with an increase in the odds of final victory by as much as 31.7%, and performing one extra sprint in the first half—by as much as 8.6%. Thus, the appropriate selection of the training centres can directly affect the match outcome.

As Racinais et al. (2008) observe, thermal stress is a serious problem for athletes because it not only significantly impairs their particular physical activities but also decision-making accuracy. Thermal stress can lead to a decrease in psychomotor skills, reduction in speed and precision of movement, and impaired concentration (Mohr et al. 2012; Nybo et al. 2014). The present study partially confirms this by reporting the number of passes as significantly lower in TS → TS than in TS → NTS. However, no significant relationships were found between the number of shots and pass accuracy and biometeorological conditions. Nassis et al. (2015) produced different findings in their analysis of matches of the World Cup in Brazil in 2014. They found that the number of passes did not differ, but the rate of successful passes was significantly higher (*p* < 0.05) under high (77%) than under low (74%) environmental stress. These differences may result from the use of different methods of thermal stress assessment, i.e., the UTCI (present study) and the WBGT (Nassis et al. 2015). Both indices are weakly correlated with each other. The UTCI is more sensitive than the WGBT, and the greatest differences can be observed at elevated temperatures (Błażejczyk et al. 2012). However, it is also worth emphasizing that the percentage of successful passes at the World Cup in Russia was much higher and ranged from 82% (TS → TS) to 84% (NTS → TS). However, the differences between successful pass rates were not statistically significant. As part of the match strategy related to biometeorological conditions, professional soccer players are able to maintain their technical activities at a high level by modifying the intensity of covered distances (Nassis et al. 2015). Moreover, a player with a body temperature below 39 °C, who is properly hydrated, will not experience any significant impairment of their performance skills (Mohr et al. 2010).

In the context of players’ adequate hydration, the present study constituted an attempt to confront theoretical models for determining physiological parameters with other published data recorded during the game. Thermal stress has many negative consequences for the body, e.g., blood flow direction towards the skin surface (with the consequential increase in heart rate) and transition from aerobic to anaerobic metabolism. This leads to the faster depletion of muscle glycogen, followed by fatigue and worse performance (Grantham et al. 2010). Due to the fact that physiological reactions of the body determine the players’ activity and efficiency on the pitch, it appears necessary to assess players’ physiological parameters together with the parallel use of kinematic tests. The ideal setup would be carrying such measurements during actual games, but unfortunately, it is not possible during high-stakes World Cup matches. Therefore, researchers are left only with theoretical models for determining players’ physiological parameters, whose reliability should be constantly tested, modified, and improved.

The present study indicates that both Heart Rate Effort and Evaporative Water Loss are the lowest in the NTS → NTS and the highest in the TS → TS condition ranges. This is consistent with the normal response of the body to adverse biometeorological conditions (Kurdak et al. 2010; Mohr et al. 2012). It is worth emphasizing, however, that similarly to physical activities, the HR-E is significantly lower for teams preparing in NTS in both NTS and TS matches. The EVL is also significantly lower, if teams prepare in NTS, especially, if an upcoming match is played in TS. Thus, physiological indices also show the positive effect of selecting training centres where there is a low risk of thermal stress.

The HR-E and EVL values calculated for the purposes of this study are yet another issue. They are significantly underestimated compared to studies published so far. Depending on their position on the pitch, elite players can undertake an activity at 70–75% VO_2_ max and 80–90% HR max (Stolen et al. 2005; Bangsbo et al. 2006; Rebelo et al. 2014). However, the present study showed the players’ heart rate between 128 and 140 BPM. The average evaporative water loss in elite players during a 90-min match at 32 °C is 2.2 l (Shirreffs 2010), at 26 °C about 2.0 l (Maughan et al. 2004) and at 5 °C 1.7 l (Maughan et al. 2005). Our research indicates the players’ EVL between 523 and 793 [g/h]. Most likely, such low values result from the workload for players of 400 W/m^2^ adopted in the literature (Fanger 1970; ISO 8996). Hayes et al. (2014) claim that in unfavourable biometeorological conditions, there is a tendency to increase energy expenditure, which is especially associated with intensive, short-term exercise. This type of activity is characteristic for soccer, e.g. over 600 accelerations per a Bundesliga match (Konefał et al. 2019b). Dehydration above 2% of body weight has a detrimental effect on players’ cognitive functions and physical and intellectual abilities, and it inhibits the body’s thermoregulatory mechanism (de Freitas and Ryken 1989; Nielsen 1996; Casa 1999; Heat-waves… 2004; Kurdak et al. 2010; Shirreffs 2010). Therefore, in order to improve the game effectiveness, it is worth identifying the values of this parameter in players competing in top-level soccer tournaments. However, it should be emphasized that soccer players’ physiological responses to adverse biometeorological conditions are highly individual (Ozgunen et al. 2010; Arsac et al. 2013).

As Hosokawa et al. (2018) claim, the wet-bulb globe temperature might exceed 30 °C in 40 to 50% of the late mornings and early afternoons during the 2020 Olympic soccer tournament in Japan. This means that the next major football tournament after South Africa and Brazil will take place in unfavourable biometeorological conditions. The 2022 World Cup in Qatar has been moved to the autumn season, which will make it less thermally troublesome for players. Data analysis and discussion in the present study provide the basis for a serious consideration of climate as one of the most important factors for both sports and health reasons. It seems that the search for optimal solutions for soccer players preparing for tournaments is highly significant, and the World Cup or the Olympic Games organizers should encourage the relevant football authorities (i.e. FIFA and national football associations) to plan the venues and dates of tournaments in locations where the risk of thermal stress is minimized. National team training staffs should also ensure that the climatic conditions at the selected training centres are thoroughly verified.

## Conclusions

The climatic conditions of soccer training centres and the conditions in which matches are played must be considered in planning preparations for future World Cup tournaments, especially those in hotter countries. Situations where the climatic conditions at the training centres indicate no thermal stress (UTCI between 9 and 26 °C) are more beneficial for increasing only the physical activity (total distance covered and number of sprints) of players.

Theoretical models for determining players’ physiological parameters used in the present study reduce the values of heart rate effort and evaporative water loss indicated in scientific literature. In theoretical models, it is necessary to verify players’ adopted energy load values during soccer matches, especially in unfavourable biometeorological conditions. The players’ reactions to HR-E and EVP changes in climatic conditions are, however, correct.
